# Fighting Fire with Fire: Exosomes and Acute Pancreatitis-Associated Acute Lung Injury

**DOI:** 10.3390/bioengineering9110615

**Published:** 2022-10-26

**Authors:** Qi Yang, Yalan Luo, Bowen Lan, Xuanchi Dong, Zhengjian Wang, Peng Ge, Guixin Zhang, Hailong Chen

**Affiliations:** 1 Department of General Surgery, The First Affiliated Hospital of Dalian Medical University, Dalian 116011, China; 2 Institute (College) of Integrative Medicine, Dalian Medical University, Dalian 116044, China; 3 Department of Traditional Chinese Medicine, The Second Affiliated Hospital of Dalian Medical University, Dalian 116023, China; 4 Laboratory of Integrative Medicine, The First Affiliated Hospital of Dalian Medical University, Dalian 116011, China

**Keywords:** exosome, acute pancreatitis, acute lung injury, non-coding RNA, targeted therapy, diagnosis

## Abstract

Acute pancreatitis (AP) is a prevalent clinical condition of the digestive system, with a growing frequency each year. Approximately 20% of patients suffer from severe acute pancreatitis (SAP) with local consequences and multi-organ failure, putting a significant strain on patients’ health insurance. According to reports, the lungs are particularly susceptible to SAP. Acute respiratory distress syndrome, a severe type of acute lung injury (ALI), is the primary cause of mortality among AP patients. Controlling the mortality associated with SAP requires an understanding of the etiology of AP-associated ALI, the discovery of biomarkers for the early detection of ALI, and the identification of potentially effective drug treatments. Exosomes are a class of extracellular vesicles with a diameter of 30–150 nm that are actively released into tissue fluids to mediate biological functions. Exosomes are laden with bioactive cargo, such as lipids, proteins, DNA, and RNA. During the initial stages of AP, acinar cell-derived exosomes suppress forkhead box protein O1 expression, resulting in M1 macrophage polarization. Similarly, macrophage-derived exosomes activate inflammatory pathways within endothelium or epithelial cells, promoting an inflammatory cascade response. On the other hand, a part of exosome cargo performs tissue repair and anti-inflammatory actions and inhibits the cytokine storm during AP. Other reviews have detailed the function of exosomes in the development of AP, chronic pancreatitis, and autoimmune pancreatitis. The discoveries involving exosomes at the intersection of AP and acute lung injury (ALI) are reviewed here. Furthermore, we discuss the therapeutic potential of exosomes in AP and associated ALI. With the continuous improvement of technological tools, the research on exosomes has gradually shifted from basic to clinical applications. Several exosome-specific non-coding RNAs and proteins can be used as novel molecular markers to assist in the diagnosis and prognosis of AP and associated ALI.

## 1. Introduction

Acute pancreatitis (AP) is a frequent occurring, acute abdominal illness. The majority of patients present with mild AP, which may spontaneously resolve. Nonetheless, approximately 20% of patients report severe AP (SAP) that is fast progressive and aggressive [1,2]. Acute lung injury (ALI) is a life-threatening condition characterized by diffuse interstitial and alveolar edema resulting from the damage of pulmonary microvascular endothelial cells (PMVECs) and alveolar epithelial cells (AECs) [3,4]. ALI and its severe form, acute respiratory distress syndrome (ARDS), are among the most prevalent consequences of SAP and are leading causes of mortality in SAP patients [5].

New insights into the pathogenesis of AP and associated ALI have emerged in recent years. The systemic inflammatory response (SIRS) caused by the abnormal activation of pancreatic enzymes, mitochondrial dysfunction, impaired autophagy, endoplasmic reticulum stress, programmed cell death, intestinal mucosal barrier damage, and bacterial translocation are the initiating factors of multiple organ dysfunction syndromes (MODS) in AP [6]. Key molecules causing pulmonary air–blood barrier disruption and alveolar edema include pancreatic, intestinal, and liver-derived non-coding RNAs (ncRNAs), damage-associated molecular patterns (DAMPs), and pathogen-associated molecular patterns (PAMPs). Cross-signaling between immune cells (such as neutrophils, macrophages, and T cells) and parenchymal cells (such as acinar cells, intestinal epithelial cells, PMVECs, and AECs) is a crucial mechanism for maintaining the AP cytokine storm [7]. However, the molecular network of intercellular communication is intricate and requires immediate clarification. Exosome-related research has expanded quickly in recent years. The basics of exosomes, including the biogenesis, processes of secretion, and cargo they carry, have been steadily uncovered [8,9]. We hypothesize that exosomes may be a key mediator of communication between immune cells and parenchymal cells, mediating the local and systemic inflammatory response during AP.

Extracellular vesicles (EVs), which include various types of vesicles such as exosomes, microvesicles, and apoptotic vesicles ranging from 30 nm to 10 µm in diameter, were first discovered by Erwin Chargaff and Randolph West using high-speed centrifugation; they detected a platelet-free clotting component in plasma [10,11]. Later, Johnstone et al. discovered and suggested EVs as a cellular waste disposal mechanism in the culture supernatant of sheep erythrocytes [12]. Since the 21st century, numerous studies have reported the importance of exosomal cargo and its function as a unique biological device [13,14]. The biogenesis of exosomes involves both production and secretion. The process of exosome production mainly consists of two invaginations of the plasma membrane and the formation of a multivesicular body (MVB) containing intraluminal vesicles (ILVs). The MVBs bind to lysosomes for degradation and fuse again with the cytoplasmic membrane to release ILVs, completing the exosome release process. Exosome stability is a major contributing factor to their widespread acceptance. The first thing to know about exosomes is that they are very tiny and can thus easily pass through both blood vessels and the extracellular matrix. Since they are released by one’s own cells, monocytes and macrophages cannot phagocytose exosomes. Furthermore, exosomes can circulate freely in bodily fluids because CD55 and CD59 on their surface protect them from being altered by opsonin and coagulation factors [15]. As a result of their unique vesicular structure, the contents of exosomes may be kept reasonably stable for up to a few weeks [16]. Kalra et al. [17] showed by proteomic analysis that the protein composition of plasma exosomes is stable for up to 90 days and may still be picked up by target cells and exert biological effects. In addition, it has been observed that plasma exosomal microRNAs (miRNAs) are relatively stable, even when kept in various storage settings [18]. When stored at −20 degrees Celsius for 5 weeks, the overall quantity of exosomal miRNA remained almost the same, with very modest variations in the amounts of specific miRNAs. Thus, when exosomes are picked up by the recipient cells and internalized, they contain cargo that can impact the recipient cell function through triggering intracytoplasmic signaling pathways [19]. Specifically, exosome-derived nucleic acids and proteins are widely involved in physiopathological processes such as the inflammatory response, infection, and immune regulation, affecting the development of AP [20].

This paper sheds light on the AP-mediated cytokine storm by describing the signaling regulatory actions of exosomal proteins and nucleic acids. We will also show that exosomes, which are found in all biological fluids, including blood, bronchoalveolar lavage, urine, and pancreatic fluid, may be used as diagnostic and prognostic indicators for AP. Furthermore, exosomes are stable, non-toxic liposomes that may escape immune detection and be absorbed by recipient cells. With the growth of exosome research and the implementation of new experimental procedures, it is anticipated that exosomes will also be exploited for medication delivery in clinical study.

## 2. Exosomes and AP-Associated ALI

The molecular mechanisms involved in AP-associated ALI that lead to SIRS and diffuse alveolar damage have been studied in detail. Multiple signaling pathways are engaged during AP. To summarize, the activation of the cytokine storm is caused by the upregulation of extracellular mediators such as DAMPs, histones, and ncRNAs during AP. Intriguingly, emerging research has shown that the pancreas–lung axis [6] and the gut–lung axis [21] may mediate the cytokine storm in AP-associated ALI, and that exosomes may be major carriers of extracellular mediators transported along the signaling axis.

We hypothesize that the pancreas–lung axis is a putative signaling pathway for AP-associated ALI based on the findings of many investigations. Zhu et al. discovered that plasma exosomal miR-216a was considerably elevated in AP patients with ALI compared to AP patients without ALI [22]. Exosomal miR-216a seems to be a particular modulator of inflammation in AP-induced ALI. As shown in animal investigations, miR-216a expression was undetectable in all organs save the pancreas, including the lung, gut, heart, and kidney. It is possible that exosomal miR-216a is pancreas-specific. Moreover, exosomal miR-216a enhanced the permeability of pulmonary microvascular endothelial cells, which was linked with the degree of ALI during AP. Xu et al. discovered that cold-inducible RNA-binding protein (CIRP) may play a crucial role in alveolar macrophage (AM) pyroptosis as well as neutrophil recruitment during AP-associated ALI [23]. The level of CIRP was found to be enhanced in the pancreatic tissue, serum, and lung tissue of AP rats by Xu and his colleagues. Interestingly, immunohistochemical staining revealed that pancreatic islet cells may be the predominant cell type that secretes CIRP, which may be an additional inflammatory mediator secreted by injured pancreatic tissue that induces ALI. In addition, Murao et al. discovered that CIRP may persist extracellularly as exosomes and mediate inflammation during sepsis. Thus, we hypothesized that exosome-loaded inflammatory mediators, such as CIRP and miR-216a, delivered along the pancreas–lung axis mediate the development of AP-associated ALI.

The gut–lung axis is a commonly recognized pathophysiological signal of crosstalk between intestinal and pulmonary diseases. In the case of AP-associated ALI, intestinal damage and its subsequent response has an “amplifier” effect [24]. Firstly, intestinal barrier damage and increased intestinal permeability are prevalent in AP patients and models generated by a variety of causes [25,26]. Secondly, the intestinal barrier is a factor that exacerbates the inflammatory response to SIRS [27]. After intestinal barrier damage, the most direct consequence may be the “second strike” of intestine-derived endotoxins entering the circulation and lungs through the portal vein or mesenteric lymphatic system [28,29]. On the other hand, SIRS may be promoted by exosomes released from the injured gut during AP. Under physiological conditions, intestinal epithelial cells (IECs) or DC-derived exosomes carrying transforming growth factor-β, MHC class I and II complexes and co-stimulatory molecules coordinate the regulation of intestinal immunity and maintain immune homeostasis. However, miRNAs such as miR-122a and miR-29a released from damaged IECs can exacerbate intestinal barrier damage and increase intestinal permeability [30].

**Figure 1:**
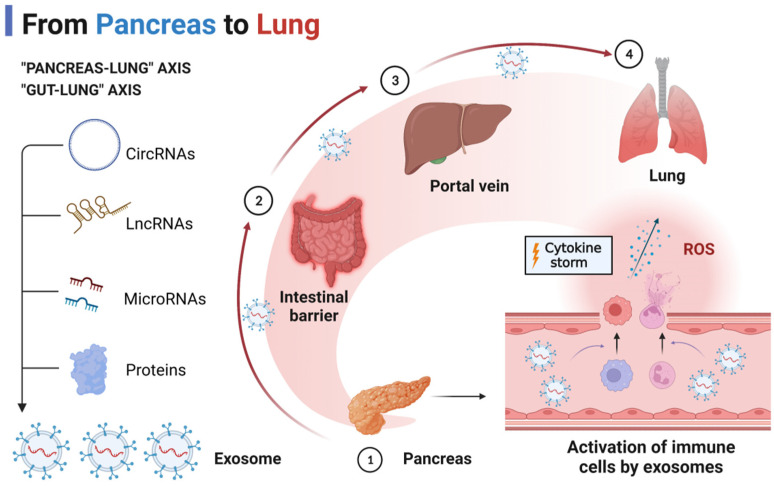
The “pancreas–lung” axis and “gut–lung” axis in AP-associated ALI.

Thus, we hypothesize that the pancreas and gut release exosomes containing pro-inflammatory mediators during AP, with the pancreas–lung axis and the gut–lung axis serving as the primary delivery pathways (Figure 1).

## 3. Exosome-Specific ncRNAs

### 3.1. LncRNAs

LncRNAs, or long non-coding RNAs, are a subset of ncRNAs that are longer than 200 nucleotides and are synthesized in a manner analogous to mRNA. First dismissed as meaningless “transcriptional noise” [31], lncRNAs have now been shown to play essential roles in various biological processes. A growing body of evidence indicates that lncRNAs may interact with RNA, DNA, and proteins. In particular, lncRNAs can regulate gene expression at the epigenetic, transcriptional, and post-transcriptional levels and even directly regulate protein activity [32,33,34,35]. The lncRNAs may also take part in chromatin modification [36]. Many lncRNAs have been implicated in pathophysiological processes such as cancer [37], inflammation [38], hematopoiesis [31], and metabolism [39]; the interest in lncRNAs has grown with the advancement of high-throughput technology.

#### 3.1.1. MALAT1

As one of the first lncRNAs discovered, metastasis-associated lung adenocarcinoma transcript 1 (MALAT1) has been linked to the pathophysiology of inflammatory illnesses, metabolic diseases, and cancer [40,41,42]. As discovered by Gu et al. [43], the early inflammatory response of acinar cells may include a “MALAT1-miR-194-yes-associated protein 1 (YAP1)” mutual feedback loop. MiR-194 is a protective microRNA that dampens cerulein-induced inflammation in AR42J cells (pancreatic acinar cells) by targeting the YAP signaling pathway. It is interesting to note that, in vitro, YAP1 and MALAT1 enhance each other, whereas MALAT1 and miR-194 counteract each other [43]. It has also been established that the MALAT1/miR-181a-5p/high-mobility group box 1 (HMGB1) axis plays a role in macrophage polarization during AP [44]. First, the researchers found elevated levels of MALAT1 in EVs taken from the plasma of AP patients compared to those found in healthy participants. MPC-83 cells (mouse pancreatic acinar cells) were encouraged to generate MALAT1-carrying EVs in response to cerulein, providing further evidence that the MALAT1-carrying EVs observed in AP patients’ plasma likely originate from inflamed acinar cells. Mechanistic studies demonstrated that MALAT1 upregulates HMGB1 (a notorious DAMP) expression by competitively binding to miR-181a-5p, stimulating the toll-like receptor 4 (TLR4)/nuclear factor kappa B (NF-κB) signaling pathway and promoting M1 macrophage polarization [44]. It is noteworthy that hepatocytes, similar to acinar cells, also emit exosomes containing MALAT1. Risk factors for AP include the presence of non-alcoholic fatty liver disease. The authors hypothesized that hepatocyte injury and the subsequent release of exosomes would foster the progression of AP [45]. Exosome-specific MALAT1 was secreted by damaged hepatocytes, which exacerbated inflammation in acinar cells by downregulating autophagy and upregulating the Hippo–YAP pathway [45].

In addition to AP, MALAT1 expression was elevated in the plasma of sepsis and ARDS patients [46,47]. The expression of MALAT1 was strongly connected with illness severity, organ damage, and death, and its diagnostic performance in patients with sepsis was found to be excellent (AUC = 0.931) by Lu et al. [46]. MALAT1 levels in plasma and peripheral blood mononuclear cells were considerably more significant in ARDS patients compared to the control group. It was also shown that MALAT1 levels in plasma exosomes were higher than in the control group, though this difference did not reach statistical significance [47]. All studies have consistently linked MALAT1 to worse outcomes in ALI/ARDS from a mechanical standpoint. Moreover, the knockdown of MALAT1 expression significantly inhibited the apoptosis of alveolar epithelial cells, disruption of the endothelial barrier, and inflammatory signaling pathways in ALI.

#### 3.1.2. TUG1

LncRNA taurine upregulated gene 1 (TUG1) consists of 7598 nucleotides and was first discovered in the retinal cells of neonatal mice. Retinal development in mice is hampered when TUG1 is silenced [48]. TUG1 expression was markedly downregulated in lung tissue and airway epithelial cells in sepsis-induced-ALI animals. By blocking miR-494, TUG1 protected the airway epithelial cells and reduced lung inflammation [49]. If pulmonary microvascular endothelial cells are under assault, TUG1 may potentially serve as a protective factor. In particular, increasing TUG1 expression suppressed lipopolysaccharide (LPS)-induced apoptosis and inflammation in endothelial cells. Overexpressing miR-34b-5p in rescue tests decreased TUG1’s protective impact on endothelial cells. GRB2-associated binding protein 1, positively correlated with TUG1, was negatively regulated by miR-34b-5p and, similarly, exerted a protective effect [50]. EVs-specific TUG1 is also protective against sepsis-induced ALI. Ma et al. [51] reported that EVs released from endothelial progenitor cells were rich in TUG1, promoting tissue repair, and attenuating vascular injury by inducing macrophage polarization towards the M2 type. Mechanistically, TUG1 upregulates silent information regulator 1 (SIRT1) expression by competitively binding miR-9-5p. The latter is known as an antioxidant and anti-inflammatory deacetylase. In contrast to ALI, TUG1 and exosomal TUG1 expression was stimulated to be upregulated in AR42J cells and was significantly associated with Treg cell differentiation. The overexpression of TUG1 promotes LPS/cerulein-induced apoptosis and inflammation in AR42J cells [52]. According to recent research, lncRNA TUG1 may play opposing roles in AP and ALI. As there are no experimental investigations on the impact of exosomal TUG1 produced by acinar cells on lung parenchymal cells, such as epithelial and endothelial cells, this piqued our curiosity.

#### 3.1.3. Others

Patients with AP or ARDS may potentially benefit from testing for a variety of additional lncRNAs that have been proven to have diagnostic utility. Patients with AP have an elevated plasma plasmacytoma variant translocation gene 1 (PVT1) expression compared to healthy volunteers; this elevated expression is correlated with various clinical characteristics and has a substantial predictive value for mortality (AUC = 0.838) [46]. Similarly, PVT1 can be an independent risk factor for patients with ARDS and can help to assess the severity of ARDS and predict mortality [53]. In addition, exosome-specific PVT1 has been shown to have diagnostic relevance in human disease [54]. As a result, we have become more curious about exosome-specific PVT1 and its function in AP and ALI. Other lncRNAs, such as FBXL19-AS1 [55] and lnc-ITSN1-2 [56], have also been shown to be helpful in the diagnosis of AP and the evaluation of prognosis. It is essential to investigate whether such lncRNAs are present in exosomes and, if so, what role they play in AP and the accompanying ALI.

### 3.2. MiRNAs

According to the current data, microRNAs (miRNAs) are the primary form of RNA delivered by exosomes. MiRNAs are small ncRNAs that are highly conserved in organisms and are approximately 20–24 nucleotides in length [57]. In recent years, miRNAs have been shown to regulate gene expression at the post-transcriptional level by pairing with mRNA 3′-untranslated regions of target genes [58]. The functions of miRNAs were more than imagined [59,60]. At present, the synthesis process of miRNA in animals is well understood. MiRNA is first transcribed in the nucleus by RNA polymerase II into pri-miRNA, which is about 300–1000 bases in length. Pri-miRNA is cleaved in the nucleus by RNase III-Drosha into precursor miRNA (pre-miRNA), at approximately 70 bases in length. Subsequently, the pre-miRNA is transferred from the nucleus to the cytoplasm by the action of the transporter protein exportin 5 and further cleaved by RNase III-Dicer into a mature double-stranded miRNA (mature miRNA) of approximately 20–24 nt in length [61,62]. The mature double-stranded miRNAs form RNA-induced silencing complexes with Argonaute proteins to degrade target gene mRNAs or play a biological role in translation inhibition [63]. MiRNAs have become a hot research target as regulators of gene expression and biomarker candidates. MiRNAs in exosomes are important in disease development, diagnosis, etc. [64]. The discovery of exosomal miRNAs in AP, a common and dangerous acute abdominal disease, provides new ideas on the pathogenesis and diagnosis of AP and has some reference value.

#### 3.2.1. MiR-155

MiR-155 is a miRNA encoded by a non-coding B cell integration cluster, with two mature forms, miR-155-3p and miR-155-5p. MiR-155 has a crucial regulatory function in the organism’s innate immune and inflammatory response [65,66]. The miR-155 level is often increased in the presence of severe disease [67,68]. The expression of miR-155 in the peripheral blood of AP patients is, however, debatable. Lin et al. discovered that the expression of miR-155 was considerably higher in the serum of AP patients and strongly linked with the Ranson score and APACHE II score [69]. Wang et al. also found a correlation between higher miR-155 levels and the severity of AP patients [70]. Miao et al. found that the level of plasma-derived exosomal miR-155 in AP-associated ALI was significantly higher than in AP without ALI. In comparison, the exosome-specific miR-155 level in the AP without the ALI group was significantly higher than that in healthy volunteers [71]. However, Hu et al. discovered considerably lower miR-155 levels in the blood of AP patients than in healthy participants [72]. The cause for this opposite outcome is, as yet, unclear. More clinical research with large sample sizes is needed to assess miR-155 levels in AP patients.

MiR-155 appears to be consistently highly expressed in the pancreas, gut, lung, and peripheral blood of AP models. Inhibition of miR-155 alleviates intestinal barrier damage [73], immune imbalance [70], and impaired autophagy [74,75] induced by AP. For exosome-specific miR-155, researchers discovered that plasma-derived exosomes were enriched with inflammatory miR-155, whereas ascites-derived exosomes were not [76]. Exosome-specific miR-155 that the liver has activated may reach the alveoli and contribute to the development of ALI. Specifically, exosome-specific miR-155 activates alveolar macrophages and triggers NOD-like receptor protein 3 (NLRP3)-dependent pyroptosis [77]. The inhibition of MiR-155 alleviates ALI by inhibiting macrophage proliferation [78], antagonizing nuclear factor kappa beta (NF-κB) signaling [79], and modulating neutrophil extracellular trap formation [80] in the lungs.

#### 3.2.2. MiR-21

The miR-21 gene is found in the 10th intron region of the transmembrane protein 49 gene on chromosome 17 q23.2. MiR-21, unlike other miRNAs, has an independent promoter region and is regulated by transcription factors, including AP-1, STAT3, and p53 [41]. In research including 164 patients, miR-21-3p levels in AP groups were considerably higher than in the healthy control group [81]. The level of miR-21-3p was considerably more significant in the SAP group compared to the MSAP and MAP groups. In patients with autoimmune pancreatitis, the level of extracellular vesicle (EV)-specific miR-21-5p in the plasma was considerably higher than in patients with chronic pancreatitis and healthy volunteers [82]. Hu and colleagues discovered, similar to miR-155, that AP patients had lower levels of miR-21 than healthy volunteers [72]. Therefore, the level of miR-21 in the circulation of AP patients requires additional investigation.

MiR-21 is said to favor the AP procedure. Stimulation of acinar cells with cerulein and taurolithocholic acid 3-sulfate induced significant changes in miRNA expression profiles, with miR-21-3p being significantly elevated [54]. The mouse AP model induced by cerulein and L-arginine also confirmed the high level of miR-21-3p in the pancreatic tissue [83]. Mechanistically, miR-21-3p exacerbated inflammation and lung injury by activating transient receptor potential signaling pathways in SAP rats [84]. Knockdown of miR-21 decreased AP-associated ALI by reducing trypsinogen activation [85] and inhibiting the release of HMGB1. Intriguingly, miR-21-5p may prevent lung damage. In AEC cells, the overexpression of miR-21-5p inhibited hyperoxia-induced apoptosis [86] and mitochondrial damage [87]. Exosomes generated from mesenchymal stromal cells decreased ischemia/reperfusion-induced ALI in an miR-21-5p-dependent manner [88].

#### 3.2.3. MiR-216a

Extensive research has been conducted on the function of miR-216a in diagnosing AP [89,90,91]. The miR-216a level was elevated in the plasma and mesenteric lymph after induction of AP in rats and mice and was positively correlated with the severity of the disease [92]. In an AP dog model, elevated miR-216a levels were substantially linked with the extent of tissue damage and showed a more dynamic response than amylase and lipase [93]. According to a clinical investigation, the plasma miR-216a level in SAP patients was considerably higher than in mild AP (MAP) and MSAP patients, but there was no difference between MAP and MSAP patients [94]. This indicates that miR-216a may serve as a biomarker for the early detection of SAP. Notably, miR-216a was tightly linked to SAP-induced liver damage. In SAP patients with liver injury, miR-216a levels in the peripheral blood were considerably higher than in SAP patients without liver injury [95]. Furthermore, Zhu et al. examined the differences in plasma-derived exosomal miR-216a levels in patients with AP-associated ALI, patients with AP without ALI, and healthy volunteers. They found that exosome-specific miR-216a levels were significantly higher in patients in the AP-associated ALI group than in the AP without ALI group, which in turn had significantly higher exosome-specific miR-216a levels than in healthy volunteers [22]. These results suggest that miR-216a is present in plasma-derived exosomes and plays a vital role in the process of AP-associated ALI. Mechanistic experiments confirmed the role of miR-216a as a risk factor for PMVEC injury in AP. Overexpression of miR-216a increases endothelial cell permeability by altering the expression of tight junction proteins and is associated with the development of lung injury. However, Fan et al. [96] offered an alternative viewpoint. They discovered that miR-216a overexpression in ALI animals dramatically decreased serum inflammatory factor levels and improved lung permeability. The miR-216a target Janus protein tyrosine kinase 2 dramatically reduced ALI by regulating the NF-κB signaling pathway [96]. Thus, many more experiments are needed to prove the function of miR-216a in AP and associated ALI.

#### 3.2.4. Others

A recent clinical study reported that seven exosome-specific miRNAs might be potential biomarkers for the diagnosis of patients with SAP [97]. Among them, the levels of exosome-specific miR-583 and miR-603 were strongly correlated with the severity of AP [97]. MiR-583 and miR-603 are considered to be tumor suppressors, and their related studies are mainly focused on tumors such as breast cancer [98], hepatocellular carcinoma [99], and colorectal cancer [100]. There are no mechanistic studies on the association of miR-603 with AP or SIRS. Exosome-specific miR-192-5p levels were downregulated in mild AP patients. This result is similar to that of previous studies. Patients with AP with nonalcoholic fatty liver disease had significantly lower circulating miR-192-5p levels than the healthy control group [101]. Furthermore, the level of exosome-specific miR-122-5p was upregulated only in the SAP group but not in the MAP group [97]. Interestingly, miR-122-5p has previously been shown to be associated with lipopolysaccharide (LPS)-induced ALI. Knockdown of miR-122-5p helps to attenuate the damage of PMVECs and AECs during ALI. Thus, miR-122-5p may be the essential medium for SAP-associated ALI [102,103]. This, of course, needs to be confirmed by further studies.

**Figure 2:**
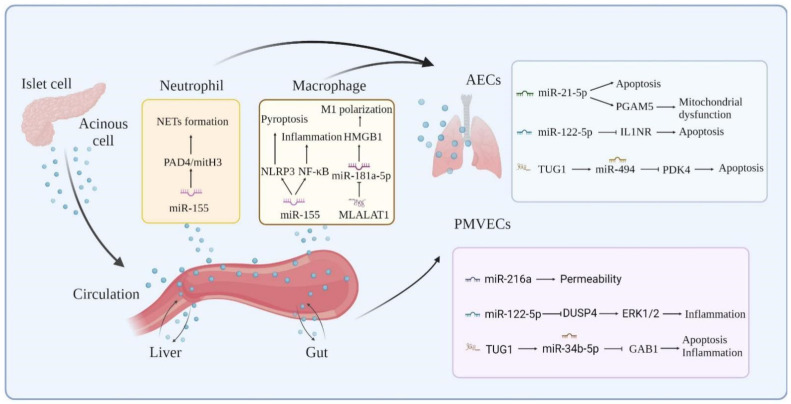
Molecular mechanisms of exosome-ncRNAs in AP-associated ALI.

The role of exosome-specific ncRNAs in SIRS is a rapidly developing area of study in AP. Synergistic interactions between neutrophils, macrophages, platelets, endothelial cells, and AECs create the cytokine storm of AP and associated ALI. Several exosome-specific lncRNAs and miRNAs have been shown to have an altered expression in AP and ALI (Table 1), and these genes play essential roles in the AP-induced inflammatory cascade response, AM activation, increased endothelial cell permeability, and alveolar epithelial cell injury by targeting and regulating downstream genes and associated signaling pathways (Figure 2).

## 4. Exosome-Specific Proteins

Proteins, in addition to ncRNAs, are the most common exosomal cargo. Exosome-specific proteins are mostly found on the vesicle surface and in the endolumen. Membrane transport and fusion proteins, transmembrane proteins, and marker proteins such as CD63 and CD9 are found on the vesicle surface, while heat shock proteins, signal transduction proteins, and backbone proteins are found in the vesicle lumen. Exosome-specific proteins are physicochemically stable, less vulnerable to the extracellular setting, and may play an essential role in disease development [104,105]. 

### 4.1. HMGB1

High-mobility group box 1 protein (HMGB1) is a well-known DAMP that received its name from its high mobility in polyacrylamide gel electrophoresis [106,107]. In 2006, Yasuda et al. discovered a link between serum HMGB1 levels and disease severity in SAP patients. This work piqued the attention of academics in investigating the role of HMGB1 in AP [108]. Later, it was shown that serum HMGB1 levels in SAP patients might be an essential predictor of intestinal barrier failure and infection [109], and aid in predicting the risk of AP-related death [110]. Mechanistically, the A box of HMGB1 has an anti-inflammatory effect and inhibits AP-induced pancreatic injury [111]. In contrast, the B box has pro-inflammatory activity and is involved in the inflammatory response and organ damage in AP as a late inflammatory mediator. Specifically, to activate inflammation-related signaling pathways, promote the release of the neutrophil extracellular traps (NETs), induce cell necrosis, and amplify pancreatic inflammation, AP stimulates pancreatic acinar cells, pancreatic macrophages, and Kupffer cells to release HMGB1 rapidly [112,113,114,115]. The AP inflammatory cascade response has a refueling station in the gut. SAP patients are more susceptible to complications such as ALI/ARDS and sepsis because of disruption to the intestinal barrier [6]. HMGB1 has been implicated in SAP-induced intestinal barrier injury via the activation of the TLR4/9/NF-κB signaling pathway, disruption of intestinal flora, and disruption of intestinal tight junctions, according to several research findings [116,117,118]. Similarly, AP-associated ALI is exacerbated by HMGB1, which promotes inflammation by triggering nuclear translocation of NF-κB and inflammasome formation [119,120]. SAP may cause myocardial injury, which is an uncommon yet life-threatening consequence. Myocardial damage from SAP may be reduced if HMGB1-mediated oxidative stress is blocked [121]. Thus, HMGB1 may be a direct inflammatory cytokine that helps begin and sustain the inflammatory cascade response in the pancreas, gut, lungs, liver, and heart during acute pancreatitis.

The mechanism of HMGB1 release has recently piqued the curiosity of scientists. The main ways by which cells produce DAMPs may include PCD, and lysosomal- and exosomal-exocytosis [105]. Exosome-specific HMGB1 became a new favorite among researchers, as anticipated. In addition, in 2006, Liu et al. found that HMGB1, which was identified in the vesicles of Caco-2 cells, was strongly related to inflammation-induced epithelial hyperpermeability [122]. Later, researchers discovered that exosomal HMGB1 produced from the dysbiosis of the intestinal flora might be transported from the gut to the liver, thereby causing hepatic steatosis. This finding implies that exosomal HMGB1 may be a key modulator of the gut–liver axis [123]. The gut–liver axis is a well-known feedback mechanism in the SAP process. During SAP, high circulating levels of HMGB1 are primarily caused by the release of HMGB1 from hepatocytes activated by inflammatory mediators produced from the gut. Li et al. found that the extracellular release of hepatic HMGB1 from mice with sepsis occurs via exosomes and is dependent on the TLR4/caspase-11/GsdmD signaling pathway [124]. Despite the lack of substantial evidence, exosomal HMGB1 may have an inflammatory function in SAP. However, comparable disorders have provided us with valuable clues. We speculate that exosomal HMGB1 has promise for the investigation of SAP mechanisms and clinical diagnosis.

### 4.2. S100A8/A9

Solubility in 100% ammonium sulfate is a vital feature of the S100 protein family, consisting of calcium-binding proteins with a low molecular weight [125]. Currently, a total of 25 S100 proteins have been identified. S100A8 and S100A9, all of the S100 protein family, are involved in a wide range of cellular signaling processes [126]. Monomeric, homodimeric, and heterodimeric forms of S100A8 and S100A9 function as natural immune mediators by binding to cytoplasmic membrane receptors to sustain and increase the inflammatory response [127]. In addition, research suggests that S100A8/S100A9 may serve as a diagnostic and prognostic marker for COVID-19 [128], inflammatory diseases [129], and human cancers [130].

A clinical study including 246 patients found that plasma S100A8/S100A9 levels were more significant in patients with mild and severe AP compared to healthy volunteers [131]. At the time, researchers discovered no statistically significant difference in plasma S100A8/S100A9 levels between individuals with severe AP and those with mild AP. Interestingly, when exosomes were introduced into biological research, it was discovered that the difference in exosome-specific S100A8/A9 levels from severe AP vs. mild AP was substantial. The study by Waldron et al. was the first to give us some hints [132]. They collected plasma from 12 patients with alcoholic AP and 12 healthy controls for proteomic analysis. They identified 37 differentially expressed proteins, 31 of which were upregulated and 6 of which were downregulated. Interestingly, almost all of the upregulated proteins were associated with cellular compartments, including exosomes and extracellular spaces. Among them, S100A8, a DAMP, previously described, which is closely associated with the development of SAP, was also shown to be significantly up-regulated in the plasma of AP patients [132]. The potential of exosome-specific S100A8 in predicting the severity of SAP was also confirmed in a recent study. Exosome-specific S100A8 and S100A9 isolated from the plasma of SAP patients promoted the activation of the NF-κB signaling pathway and the level of inflammatory mediators in macrophages. Furthermore, the promotion of SIRS by S100A8 and S100A9 depends on the activation mechanism of nicotinamide adenine dinucleotide phosphate (NAPDH) oxidase. In summary, these findings suggest that exosome-specific S100A8 and S100A9 are associated with the intensity of SIRS during AP [133].

Mechanistic experiments further demonstrated that the exosome-specific S100A8/S100A9 protein, which exists as a heterodimer, may induce NF-κB activation and inflammatory factor production via the activation of NADPH oxidase. These data show a link between the severity of AP and the pro-inflammatory activity of exosome-specific S100A8/S100A9. Nesvaderan et al. developed a four-gene signature including S100A8, S100A9, matrix metalloproteinase (MMP)25, and MT-ND4L that predicts severe AP with 4% accuracy [134]. According to their findings, this gene panel might be used to detect severe AP at an early stage. To summarize, exosomal S100A8/S100A9 has a unique potential in the diagnosis of AP, and its relation to disease severity and prognosis should be explored in the future.

On the other hand, local pancreatic injury may progress to systemic inflammation and ALI through the S100A8/A9 protein. S100A8/A9 is involved in the release of NETs and the activation of neutrophils [135]. S100A9 directly impacts trypsinogen activation in pancreatic inflammation via the regulation of neutrophil infiltration. The activation of trypsinogen is well-known as a critical step in in the early stages of AP [136]. As a result, AP inflammation and S100A9 go hand in hand. When activated, intracellular protein complexes known as the NLRP3 inflammasome release IL-1β and IL-18, which are proinflammatory mediators involved in cell inflammation and pyroptosis. S100A9 caused pancreatic injury and inflammation by interacting directly with VNN1 and boosting the enormous production of reactive oxygen species (ROS), which activated NLRP3 inflammasome [137], as demonstrated by Xiang et al. In ALI, S100A8/A9 promoted neutrophil activation, damaged alveolar epithelial cells, and increased excessive inflammatory and immune responses in the airways and lung tissue. The S100A9 blockade significantly reduces the ALI induced by LPS or cercal ligation and puncture [138,139]. S100A8/A9 proteins are abundantly expressed in alveolar macrophages and intestinal epithelial cells after SARS-CoV-2 infection and may be biomarkers connecting respiratory and intestinal damage [140]. This discovery shows that the S100A8/A9 proteins may be essential mediators on the gut–lung axis, a proven bridge to severe AP-associated ALI. Therefore, exosome-specific S100A8/A9 is a crucial mediator of the inflammatory response induced by AP, leading to the development of ALI.

### 4.3. CIRP

CIRP was the first cold-shock protein discovered in mammals, and it is overexpressed in response to cold shock, oxidative stress, and hypoxia [141,142]. The function of CIRP is inextricably linked to its localization [143]. Intracellular CIRP (iCIRP) protects RNA from degradation while regulating RNA transcription and translation. Multiple biological mechanisms are regulated by iCIRP, which aids cells in responding to a wide range of physiological and pathological stimuli [144]. It turns out, however, that CIRP released from cells plays an essential role in mediating innate immunity and contributing to the inflammatory cascade reaction [142]. According to recent studies, exosome-specific CIRP may play an “amplifier” role in the progression of AP to ALI. Serum CIRP concentrations were found to be an independent predictor of major adverse events in patients with SAP in a clinical study involving 252 patients in 2017. CIRP outperforms other commonly used inflammatory markers such as procalcitonin, white blood cell count, and C-reactive protein in diagnostic performance for SAP [145]. This finding suggests that CIRP is released into the bloodstream during SAP and correlates with its severity, including SIRS, ALI, and MODS. CIRP levels in the pancreas, serum, and lungs of SAP rats were also shown to be considerably elevated by Xu et al. An antagonist of CIRP, C23, dramatically reduced the pancreatic injury and systemic inflammation produced by SAP, as well as the resulting ALI [23]. These data imply that SAP-induced systemic damage is mediated in part by CIRP. In addition, an experiment in vitro showed that CIRP may target toll-like receptor (TLR) 4 of alveolar macrophages (AMs), which activates NF-κB signaling, induces NLRP3 inflammasome-mediated pyroptosis, and amplifies the inflammatory response. CIRP suppresses M2 macrophage polarization by decreasing Ras superfamilies of small G proteins/erythropoietin receptor signaling in addition to triggering pyroptosis in AMs. During ALI/ARDS, the reduction in inflammation is greatly influenced by this inflammatory pathway [146].

Neutrophils release NETs, which are web-like extrusions that may kill microorganisms directly. NETs play a crucial regulatory function in the development of severe inflammatory illnesses [147,148]. SAP-induced MODS is hypothesized to be exacerbated by the excessive formation of NETs [149]. Intriguingly, during SAP, CIRP strongly influences the formation of NETs in the pancreas. The pancreatic injury produced by NETs was significantly decreased when CIRP was targeted [150]. In addition, CIRP targets AEC as well as macrophages and neutrophils. Triggering receptor expressed on myeloid cells-1 (TREM-1) has been shown to have a role in CIRP-induced inflammation in the AEC. The AEC is stimulated to produce high quantities of chemokines and cytokines when CIRP is binding to TREM-1 [151]. Alveoli are particularly vulnerable to the effects of this. The mechanism of CIRP’s release is also of relevance. CIRP is released after a variety of cell deaths, according to earlier research. Lin and his colleagues, on the other hand, confirmed an active release mechanism of CIRP [152]. CIRP concentrations in mouse serum exosomes rose dramatically during sepsis. CIRP is present mainly on the surface of exosomes and promotes inflammation by triggering cytokine production and neutrophil migration. As a result, we anticipate that the injured pancreas in SAP will produce exosome-specific CIRP, which may be capable of targeting the lung more effectively than free extracellular CIRP [78].

### 4.4. Histones

Histones are basic proteins in eukaryotic cells that combine with DNA to form chromatin and regulate gene expression. Histones may be transferred from the nucleus to the extracellular space in response to cell stimulation, initiating the immunological response and worsening inflammation [153,154]. There are three forms of extracellular histones: (1) Nucleosomes are formed by histones bound to DNA. (2) NETs are composed of histones, DNA, histone granule proteins, and cytoplasmic proteins, among others. (3) Free circulatory histones [155]. Indeed, EVs have been found to carry histones, adding to their widespread availability in normal and pathophysiological conditions. Nair et al. found that LPS encourages macrophages to release histone-carrying microvesicles (EVs 50–1000 nm in diameter) into the extracellular compartment. Histones, detected on the vesicle surface, interacted with TLR4 to induce an inflammatory response [156]. N-terminally cleaved histones H3 and H2A were also discovered in the exosomes [156]. Additionally, histones have been previously detected in many proteomic investigations of EVs [157,158]. Although no experimental investigations have demonstrated a direct link between histones and exosomes in AP, we believe this is simply a matter of time. Since extracellular histones are so prominent in AP pathogenesis, this makes sense.

The presence of extracellular histones, nucleosomes, and NETs in the bodily fluid of AP patients is clinically relevant. CitH3 levels were significantly higher and diagnostically helpful in patients with septic AP compared to healthy volunteers and non-septic AP patients (AUC = 0.93). Correlation analysis revealed a positive correlation between serum CitH3 and prognostic markers such as survival, duration of ICU stay, and sequential organ failure assessment scores (SOFA), suggesting that serum CitH3 may be a prognostic marker in patients with septic AP [159]. On the other hand, histones and nucleosomes in circulation are crucial for the diagnosis and prognosis of SAP patients. The diagnostic significance of plasma nucleosomes in severe AP was verified by a prospective trial of 74 patients (AUC = 0.718), which was more significant than CRP (AUC = 0.673) [160]. Researchers made the intriguing discovery that obesity increases nucleosome release in AP patients. Systemic histone release may be a significant factor to in obesity-induced SAP and its mortality [161]. Furthermore, Liu et al. found that the detection of circulating histones in plasma within 48 h after the onset of abdominal pain onset predicted persistent organ failure and death in AP patients (AUC = 0.92) [162]. They also discovered that the dead immune cells might be the primary source of circulating histones.

However, Keskin and his colleagues reached a different conclusion. They discovered that serum histone levels did not substantially vary between severe and moderate AP and had no clinical significance [163]. They proposed two possible explanations. On the one hand, the sample size was relatively modest. On the other hand, most of the included patients had biliary AP, which differed from previous studies that included a more significant proportion of AP due to alcohol abuse. Consequently, we hypothesize that the release of circulating histones during AP may be linked to the pathophysiology of inflammation. Additional clinical studies must confirm this.

The widespread organ toxicity of extracellular histones is amazing. There is a substantial relationship between circulating histones and SOFA in patients with sepsis, SAP, and severe trauma [164]. Experiments in vivo and in vitro verified the temporal association of histones with multi-organ damage, particularly cardiac and pulmonary injury [164]. The significance of histones in AP initiation was also established mechanistically. Guo et al. discovered that extracellular histones induce dose-dependent calcium oscillations [165]. Histones induce calcium oscillations in acinar tumor cells through activation of plasma membrane TLR9, which is required for trypsin activation and activation of the inflammatory signaling pathway during AP. Extracellular histones are critical mediators of AP-induced organ damage and may have clinical value in the diagnosis and prognosis of AP. Recent research has shown that histones may also be actively produced by live cells in the form of EVs [156], resulting in inflammation and sepsis. However, EV-specific histones have not been proven in AP, and additional research is needed to determine their clinical importance.

**Figure 3:**
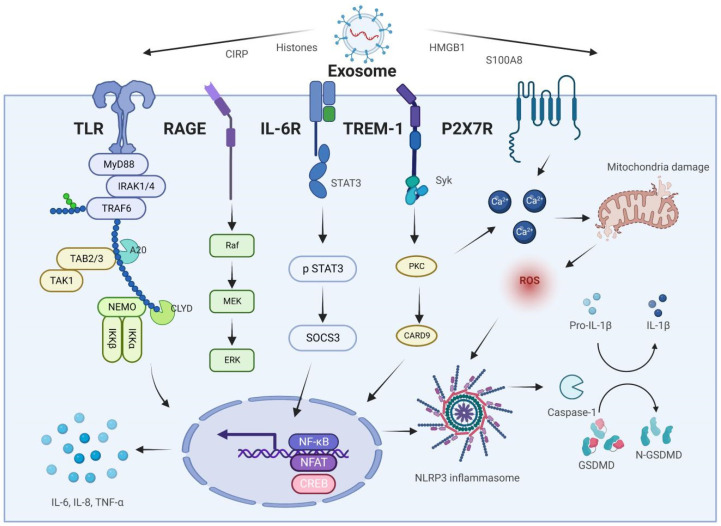
Molecular mechanisms of exosome-specific proteins in AP-associated ALI.

Exosome-specific proteins are increasingly being detected in bodily fluids and are linked to AP and associated ALI (Table 2). We hypothesize that exosomes released from the pancreas, gut, and liver, together with the proteins they contain, trigger intracellular signals that govern the receptor cell phenotype and impact the inflammatory cascade response and lung damage during AP. Exosomes transport inflammatory mediators via the pancreas–lung axis and the gut–lung axis to activate AMs, enhance their polarization toward the M1 type, and cause pyroptosis. AMs amplify the inflammatory response while also secreting exosomes, further stimulating PMVECs and AECs and disrupting the pulmonary air–blood barrier (Figure 3).

## 5. The Therapeutic Potential of Exosomes in AP and Associated ALI

Aa a double-edged sword, exosomes release both anti-inflammatory and pro-inflammatory substances into the cells to which they bind. They regulate the inflammatory cascade response during AP by selectively binding downstream molecules and modulating receptor cell activity. As a result, researchers are considering exosomes as possible therapeutic targets. The first step in the chain of AP is pancreatic acinar cell damage. Recently, in vitro experiments confirmed that exosomes produced by acinar cells were shown to drastically lower intracellular ROS and the inflammatory factor level and ameliorate pathological pancreatic injury [166]. These findings show that injured cells may be able to heal tissue damage but that certain triggering elements are required. Several in vivo and in vitro studies demonstrated emodin’s protective properties against AP-induced pancreatic injury, intestinal barrier dysfunction, and ALI [23,167]. Emodin was discovered to boost the differentiation and anti-inflammatory activity of regulatory T cells by stimulating the release of exosome-specific lncRNA taurine upregulated 1 (TUG1) from pancreatic acinar cells, hence limiting the development of AP [52]. Recent research suggests that the inflamed pancreas may release exosomes into the circulation during SAP. These exosomes have been shown to have a pro-inflammatory action. However, emodin may prevent “bad” exosomes from being secreting by acinar cells. Proteomics was employed to characterize the impact of rhodopsin on the plasma-derived exosome proteome in SAP rats. According to the results of the enrichment study, peroxisome proliferator-activated receptors (PPAR) signaling is the primary mechanism by which emodin influences the exosomal proteome. Mechanistic investigations showed that emodin protected the lungs by preventing exosome-mediated M1 polarization of alveolar macrophages via regulating PPAR signaling [168]. During AP-induced ALI, AECs are critical target cells in the lung, and their destruction directly leads to pulmonary edema and widespread alveolar injury. Evidence suggests that exosomes derived from AECs in ALI help to regulate the immune balance and the inflammatory cascade response [169]. As a natural antioxidant and anti-inflammatory agent, salidroside has been shown to protect against AP and ALI/ARDS caused by AP in mice. Activation of NF-κB, interleukin receptor-associated kinase, and tumor necrosis factor receptor-associated molecule 6 in AMs was inhibited by the salidroside-stimulated release of exosomal miR-146a from AECs and improved ALI in rats [170]. DAMPs and PAMPs activate AMs, an essential innate immune cell population during the outset of AP. They produce significant doses of inflammatory mediators that contribute to lung damage [171], unlike alveolar and alveolar epithelial cells. Similarly, pyroptotic AM-derived pyroptotic bodies increased AEC damage and vascular leakage [172]. Furthermore, the Hippo signaling pathway was activated in AECs by AMs-derived exosomal transfer RNA-derived fragments, which caused AECs ferroptosis and aided in the establishment of ALI [173]. Modulation of AMs-derived exosomes may therefore be a promising method for combating AP and associated ALI.

The therapeutic potential of mesenchymal stem cell (MSC)-derived exosomes cannot be overlooked in exosome-related treatment techniques [174,175,176]. Human umbilical cord mesenchymal stem cells (hucMSC) have been frequently recognized for their capacity for self-renewal and multilineage differentiation, particularly for the bioactive chemicals they contain for tissue repair and the management of inflammation [177,178]. Han et al. discovered that hucMSC-derived exosomes showed a remarkable tissue regeneration potential in rats suffering from traumatic pancreatitis (trauma-induced non-infectious AP) [179]. In particular, hucMSC-Evs injected intravenously could colonize injured pancreatic tissue and inhibit inflammatory response and apoptosis of acinar cells, promoting damaged tissue repair [179]. A less frequent consequence of AP is myocardial injury, which is challenging to treat and has a high death rate [180]. MSC-derived exosomes were found to upregulate vascular hemophilia factor and vascular endothelial growth factor through the activation of the Akt/nuclear factor E2 related factors 2/heme oxygenase 1 signaling pathway, which led to the amelioration of SAP-induced myocardial injury [181]. Unfortunately, the previous research did not characterize the components transported by stem-cell-derived exosomes, i.e., the molecules that exert the protective effects. Xia et al., on the other hand, discovered that adipose-derived MSC-derived exosome increased AM mitochondrial function by transferring mitochondrial components to them, which led to a reduction in pulmonary inflammation in mice [182].

Researchers have discovered that edible plants may generate nanoscale EVs with shapes and components comparable to animal exosomes [183]. Furthermore, plant-derived exosomes are biocompatible and safe to consume, with no side effects or possible toxicity [184,185]. Plant-derived exosomes may penetrate biological barriers to transport lipid-soluble and hydrophilic target molecules to tissues in vivo, increasing the target molecules’ bioavailability or effectiveness of the target molecules [186]. Ginger exosome-like nanoparticles (GELN) have been found to be taken up by lung macrophages and epithelial cells, with a preference for ACE2-positive cells. Furthermore, ginger GELN miRNA has the potential to bind to many locations in the SARS-CoV-2 virus genome and be transported to lung epithelial cells to decrease Nsp12 production and, consequently, lung inflammation [187]. As a result, plant-derived exosomes are potential AP therapeutic agents.

Exosomes maybe a therapeutic target for AP and related organ failures. Using novel medications to control the levels of lipids, proteins, and nucleic acids carried by exosomes, as well as the exosome-mediated drug delivery, offers fantastic potential to decrease the AP-induced cytokine storm. On the other hand, stem-cell-based exosome therapies have been in existence for some time and should be tested in AP clinical trials as soon as is feasible.

## 6. Exosome-Based Diagnostic Strategy

Compared with cell-free nucleic acids and proteins, exosome-specific nucleic acids and proteins are protected by a lipid bilayer and have better stability in the extracellular environment by avoiding degradation by RNA hydrolases [188,189]. In addition, exosomes are more accessible than solid biopsy samples and are present in almost all biological fluids, such as plasma, urine, saliva, ascites, breast milk, and amniotic fluid. Therefore, exosomes have been established as ideal biomarkers and have been widely used in disease diagnosis, prognosis evaluation, and treatment monitoring. The diagnostic and prognostic value of exosomes in pancreatic diseases such as pancreatic cancer and chronic pancreatitis has been widely confirmed [190,191,192,193].

**Figure 4:**
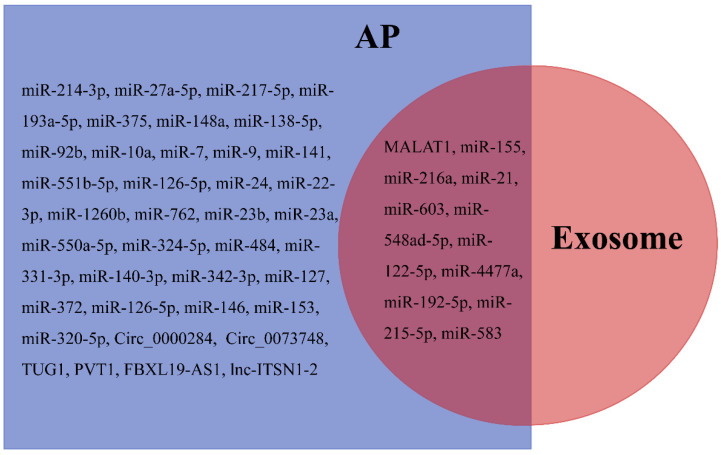
Differentially expressed non-coding RNAs in plasma/serum/mesenteric lymph/exosomes after AP.

Peripheral blood-derived exosomal miR-155, miR-216a, miR-21, (miR-603, miR-548ad-5p, miR-122-5p, miR-4477a, miR-192-5p, miR-215-5p, and miR-583), lncRNA PVT1, and MALAT1 [162] were linked with the severity of SAP and may provide new insight into the etiology of SAP and act as biomarkers of SAP. In addition, some AP serum/mesenteric lymph/plasma markers such as FBXL19-AS1 [55] and lnc-ITSN1-2 [56], miR-214-3p [194], miR-27a-5p [195], miR-217-5p [196], miR-193a-5p [197], miR-375 [198], miR-148a [199], miR-138-5p [200], miR-92b [201], miR-10a [201], miR-7 [202], miR-9 [202], miR-141 [203], miR-551b-5p [204], miR-126-5p [205], miR-24 [206], (miR-22-3p, miR-1260b, miR-762, miR-23b, miR-23a, miR-550a-5p, miR-324-5p, miR-484, miR-331-3p, miR-140-3p, and miR-342-3p [207]), miR-127 [208], miR-372 [209], miR-126-5p [210], miR-146 [211], miR-153 [212], miR-320-5p [197], Circ_0000284 [213], and Circ_0073748 [214], also exist in the exosome (Figure 4). These potential exosomal biomarkers also provide an important direction for the diagnosis and prognosis of AP in clinical applications.

Exosome-specific S100A8 correlates with the inflammatory response and predicts severity in individuals with SAP. Similarly, several free proteins and DNA are elevated in the plasma of patients with SAP, and this has implications for the diagnosis and prognostic assessment of the disease [145,215,216]. Moreover, the above substances such as HMGB1 [217], heat shock protein 70 [110], histones [162], CIRP [145], S100A12 [215], gamma-enolase [218], and mtDNA [216] have also been proven to be essential cargoes loaded by exosomes. Therefore, in the future, two areas of interest will be exploring whether the above exosome-specific cargoes can recognize AP and whether exosome-specific proteins or DNAs have better diagnostic performance than free proteins or DNAs.

In addition to blood samples, many exosomes exist in biological fluids, including urine and pancreatic juice. Urine samples are easy to obtain and non-invasive, which is desirable for both clinicians and patients. Several studies have found that nucleic acids and proteins carried by urine-derived exosomes have potential diagnostic value in pancreatic diseases [219,220,221]. In the case of AP, a 2014 study confirmed that urinary ketone bodies, glucose, plasma choline, and lipid levels were increased in patients’ urine, while levels of urinary hippurate, creatine, and plasma-branched chain amino acids decreased. A biomarker panel of guanine, hippurate, and creatine reliably identified AP with high sensitivity and specificity [222]. Later, a proteomic study confirmed that the peak intensity ratio of urinary β-2 microglobulin to saponin B has a better diagnostic performance in patients with SAP, especially with renal injury and inflammation [223]. In short, urine is also a promising biospecimen for mining AP biomarkers. Exploring the changes in exosomal cargo in urine during AP is urgent.

Pancreatic juice is secreted by pancreatic acinar cells and duct wall cells, an alkaline liquid with a strong digestibility. In terms of accuracy and the characterization that best reflects the pathological mechanisms of AP, pancreatic fluid is second to pancreatic tissue and is a biofluid superior to blood and urine [224]. In 2018, Osteikoetxea et al. found that the detection and characterization of EVs in pancreatic juice are feasible and confirmed that mucin, CFTR, and MDR1 proteins carried by pancreatic juice-derived EVs are potential biomarkers of pancreatic cancer [225]. Later, Nakamura et al. found that miR-21 and miR-15 carried by pancreatic juice-derived exosomes have the potential to diagnose patients with PDAC and CP [226]. The diagnostic value of pancreatic juice examination in pancreas-related diseases is constantly updated. The changes in the exosomal cargo of pancreatic juice in AP patients should be explored as soon as possible.

Bronchoalveolar lavage fluid (BALF), which is in direct contact with lung tissue, is an ideal biologic fluid for the diagnosis of lung diseases [227]. Previous studies have found that exosomes derived from BALF have potential diagnostic value in patients with ALI/ARDS [228], chronic obstructive pulmonary disease [229], nodular pulmonary disease [230], lung cancer [231], lung infections [232], and asthma [233]. Therefore, we speculate that BALF-derived exosomes are a potential direction for diagnosing AP-associated ALI.

Because of their stability, exosome-specific ncRNAs and proteins have been employed as biomarkers in AP and associated ALI research. Exosome isolation from blood, pleural fluid, urine, ascitic fluid, alveolar lavage fluid, and pancreatic fluid is an emerging method of fluid biopsy with broad potential clinical applications, especially for patients with AP who are experiencing multi-organ failure. Exosome-specific ncRNA and protein detection are complicated by several variables, as has been described. Different clinical investigations on the expression of a specific exosomal cargo in the bodily fluids of AP patients may find contradictory results. To further establish the sensitivity and specificity of exosomal cargoes, substantial cohort studies are still required before their use can be advocated for in clinical applications.

## 7. Conclusions

This paper sought to summarize the function of the exosomal cargo in AP and associated ALI processes. Our hypothetic pancreas–lung axis and gut–lung axis are noteworthy. After AP begins, exosomes may be released by pancreatic acinar cells, macrophages, and intestinal epithelial cells. Exosomes harboring pro-inflammatory mediators such as miR-155, miR-216a, S100A8, and CIRP are delivered through the pancreas–lung axis and gut–lung axis to the circulation and distant lung regions in order to influence the inflammatory cascade response to AP. We identified 45 ncRNAs from serum/plasma/mesenteric lymph that may be useful for the diagnosis of AP based on a comprehensive literature review spanning the last two decades. Eleven of these have been shown to be transported by exosomes in the presence of AP. Several questions have arisen, such as: (1) Does the diagnostic performance of free ncRNAs, and exosome-specific ncRNAs (the same ncRNA) vary in terms of sensitivity and specificity? (2) With so many differentially expressed miRNAs accessible, how can their value be integrated to enhance the performance of AP diagnosis? Certainly, exosome-specific proteins such as S100A8, HMGB1, and CIRP face comparable difficulties. The methods used to isolate and purify exosomes are also of interest. Numerous methods exist for isolating and purifying exosomes, but they are often somewhat involved and have their benefits and drawbacks. Furthermore, many patients find the high cost of exosome extraction unaffordable, limiting its practical use. As a result, it is crucial to establish a reliable, productive, quick, and economical method for isolating exosomes. Furthermore, exosomes have the potential to serve as a future therapy for AP. The initial objective is to identify modulatory medications, such as natural products, that are based on the pro/anti-inflammatory characteristics of the exosomal cargo. The second is loading a part of the medicine onto exosomes to enhance its bioavailability and targeting. The final objective is the source of exosomes. On the one hand, the production of exosomes derived from mesenchymal stem cells has been shown to have therapeutic value. On the other hand, plant-derived exosomes are also considered to be significant prospects.

Exosomes are involved in the development of AP and associated ALI through multiple pathways as participants in the inflammatory and immune response. Several exosome-specific ncRNAs and proteins have been studied in the diagnosis of AP, suggesting the possibility of exosomes as “novel markers”. The unique vesicular structure of exosomes has inspired researchers to develop targeted drugs. However, there is still a long way to go to promote exosomes in clinical applications, and many issues must be addressed, such as the isolation techniques, drug delivery methods, and potential toxicity. The application of exosomes in AP in the future should continue to be expanded upon to overcome these issues.

## Figures and Tables

**Table 1:** Exosome-ncRNAs in AP and ALI.

NcRNAs	Species	Targets	Expression and Role in AP	Expression and Role in ALI	Reference
MALAT1	Human, mouse, and rat	miR-194, miR-181a-5p	Up, Aggravated	Up, Aggravated	[43,44,45,47]
TUG1	Human, mouse, and rat	miR-494, miR-34b-5p, miR-9-5p	Up, Aggravated	Down, Suppressive	[49,50,51,52]
MiR-155	Human, mouse, and rat	Rictor, TAB2, SOCS1, RhoA, IL17RB, IL18R1, IL22RA2	Up/Down, Aggravated	Up, Aggravated	[70,71,72,73,74,75,76,77,78,79,80]
miR-21-3p	Human, mouse, and rat	TRPs,	Up/Down, Aggravated	Up, Aggravated	[81,72,83,84,85]
miR-21-5p	Human, mouse, and rat	Trim33, SKP2, PGAM5	Up/Down, Aggravated	Up, Suppressive	[72,82,86,87]
miR-216a	Human, mouse, and rat	JAK2	Up, Aggravated	Up, Aggravated/Suppressive	[22,92,96]

**Table 2:** Exosome-proteins in AP and ALI.

Proteins	Species	Expression and Role in AP	Expression and Role in ALI	Reference
HMGB1	Human, mouse, and rat	Up, Aggravated	Up, Aggravated	[109,112,113,114,115,119,120]
S100A8/A9	Human, mouse, and rat	Up, Aggravated	Up, Aggravated	[131,132,133,134,135,136,137,138,139,140]
CIRP	Human, mouse, and rat	Up, Aggravated	Up, Aggravated	[23,145,146,150,151,152]
Histones	Human, mouse, and rat	Up/No statistical difference, Aggravated	Up, Aggravated	[159,160,161,162,163,164,165]

## Data Availability

All data generated or analyzed during this study are included in the review manuscript.

## Abbreviations

AP	acute pancreatitis
SAP	severe acute pancreatitis
ALI	acute lung injury
PMVECs	pulmonary microvascular endothelial cells
AECs	alveolar epithelial cells
ARDS	acute respiratory distress syndrome
SIRS	systemic inflammatory response
MODS	multiple organ dysfunction syndromes
NcRNAs	non-coding RNAs
DAMPs	damage-associated molecular patterns
PAMPs	pathogen-associated molecular patterns
MVB	multivesicular body
ILVs	intraluminal vesicles
MiRNAs	microRNAs
CIRP	cold-inducible RNA-binding protein
AM	alveolar macrophage
IECs	intestinal epithelial cells
LncRNAs	long non-coding RNAs
MALAT1	Metastasis-associated lung adenocarcinoma transcript 1
YAP1	yes-associated protein 1
HMGB1	high-mobility group box 1
TLR4	toll-like receptor 4
NF-κB	nuclear factor kappa B
TUG1	taurine upregulated gene 1
LPS	lipopolysaccharide
SIRT1	silent information regulator 1
PVT1	plasmacytoma variant translocation gene 1
NLRP3	NOD-like receptor protein 3
JAK2	Janus protein tyrosine kinase 2
NETs	neutrophil extracellular traps
TLR	toll-like receptor
NAPDH	nicotinamide adenine dinucleotide phosphate
MMP	matrix metalloproteinase
ROS	reactive oxygen species
TREM-1	triggering receptor expressed on myeloid cells-1
SOFA	sequential organ failure assessment scores
PPAR	peroxisome proliferator-activated receptor
MSC	mesenchymal stem cell
GELN	ginger exosome-like nanoparticles
BALF	bronchoalveolar lavage fluid
